# Vulgar Verruca or Seborrheic Keratosis: A Clinical Challenge in Tattooed Skin

**DOI:** 10.7759/cureus.99397

**Published:** 2025-12-16

**Authors:** Carol E Marquez Maldonado, Lucia Achell Nava, Dolores M Arellano Vivero, Guadalupe Maldonado-Colin, Maria A Loredo Alanis

**Affiliations:** 1 Department of Dermatology, National Medical Center November 20, Institute of Security and Social Services of State Workers, Mexico City, MEX; 2 Department of Pathology, National Medical Center November 20, Institute of Security and Social Services of State Workers, Mexico City, MEX

**Keywords:** case report, koebner phenomenon, seborrheic keratosis, tattoo, viral warts

## Abstract

The development of verrucous lesions within tattoos poses a common diagnostic challenge, primarily between verruca vulgaris and the rarely reported phenomenon of Koebner-induced seborrheic keratosis (SK). We present the case of a 47-year-old man with a history of pemphigus vulgaris who developed multiple, brown, warty papules meticulously following the pattern of existing black ink tattoos on his trunk and shoulder. Dermoscopy revealed a cerebriform pattern with fissures, ridges, and milia-like cysts, classic for SK. Histopathological examination confirmed the diagnosis, demonstrating acanthosis, papillomatosis, hyperkeratosis, and corneal pseudocysts. No significant hypergranulosis or dilated capillaries within the dermal papillae were observed. Although HPV genotyping was not available, the overall clinicopathologic correlation supported a diagnosis of SK. This case highlights that SK may arise along tattoo tracts through trauma-related mechanisms and should be carefully distinguished from verruca vulgaris to avoid misdiagnosis and unnecessary interventions.

## Introduction

Seborrheic keratosis (SK) is one of the most prevalent benign epidermal tumors, particularly affecting middle-aged and elderly individuals [1,2]. Clinically, SK presents as well-circumscribed, verrucous papules or plaques with a stuck-on appearance [1,2]. Although the precise pathogenesis remains unclear, multiple contributing factors have been implicated, including cumulative ultraviolet radiation, oxidative stress, human papillomavirus (HPV) infection, and genetic predisposition [2,3].

The Koebner phenomenon, or isomorphic response, refers to the appearance of new skin lesions at sites of trauma in patients with pre-existing or predisposed dermatoses [4,5]. While it is classically described in psoriasis, lichen planus, and vitiligo, its occurrence in SK is exceptionally uncommon [6,7]. Several mechanisms have been proposed, including trauma-induced cytokine release, particularly interleukin-1 alpha (IL-1α), and tumor necrosis factor alpha (TNF-α), as well as keratinocyte proliferation and localized inflammatory signaling [7,8].

Tattooing, which involves repeated dermal injury and pigment deposition, may represent a potential trigger for Koebnerization in susceptible individuals [4,9]. Indeed, tattoos have been associated with various benign and infectious lesions; however, verruca vulgaris remains the most frequently reported koebnerizing lesion on tattooed skin, with numerous cases documented in the literature [4,10,11]. In stark contrast, the development of SK as a direct consequence of tattoo trauma is extraordinarily rare, with only a handful of cases reported [5,11-13]. This distinction is clinically significant, as the two entities can be challenging to differentiate visually yet require different management strategies [3,4].

We report a highly unusual case of multiple SKs arising exclusively and systematically on tattooed skin, consistent with an isomorphic response. To our knowledge, this represents one of the few documented instances of tattoo-induced Koebner phenomenon specifically manifesting as SKs. This report not only contributes to the expanding body of tattoo-related cutaneous phenomena but also highlights a critical diagnostic pitfall, underscoring the need to consider SK in the differential diagnosis of koebnerizing verrucous tattoos.

## Case presentation

A 47-year-old man with well-controlled pemphigus vulgaris and Fitzpatrick phototype IV presented for routine dermatologic follow-up. During the examination, an incidental finding of multiple cutaneous lesions was noted exclusively on tattooed skin. The patient reported that the lesions had first appeared approximately six to eight months after receiving the respective tattoos on his anterior trunk and left shoulder. They had remained completely asymptomatic (no pruritus, pain, or bleeding) and clinically stable in size and number for approximately three years prior to presentation.

Dermatologic evaluation revealed approximately 15-20 brown to dark, sharply demarcated, verrucous papules measuring 3-4 mm in diameter on the anterior trunk and left shoulder. They were distributed in a strictly linear and geometric pattern, perfectly tracing the black-ink outlines of the tattoos. No similar papules were present on the adjacent non-tattooed skin or on any other area of the body.

**Figure 1:**
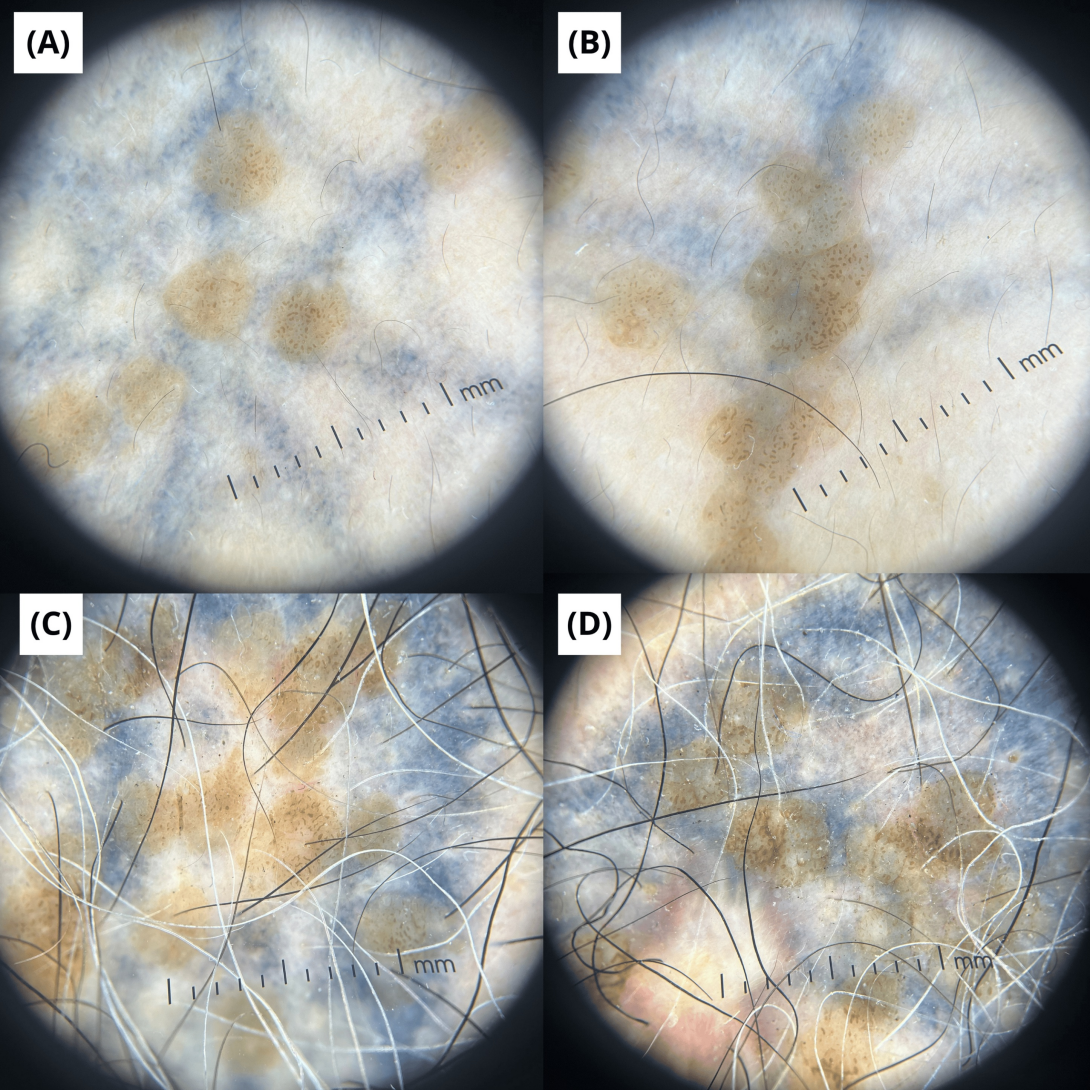
Dermoscopic findings. (A-D) Dermoscopic images (40× magnification) showing cerebriform pattern with sulci and gyri, fissures, ridges, and milia-like cysts.

Dermoscopic evaluation at 40× magnification showed a classic cerebriform pattern with sulci and gyri, fissures, ridges, and milia-like cysts. No dotted, glomerular, or hairpin vessels, or other features suggestive of a viral wart or malignancy were observed (Figure 1).

**Figure 2:**
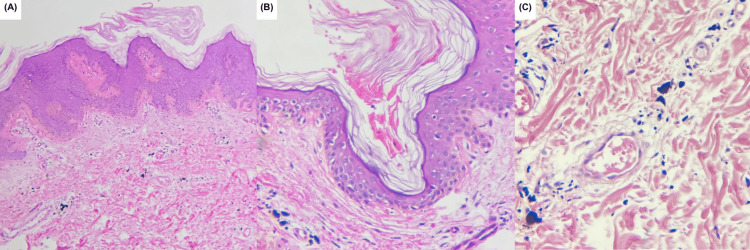
Histopathological findings. (A) Hematoxylin–eosin stain, original magnification ×10: showing acanthosis with mild papillomatosis, hyperkeratosis, and dermal pigment. (B) Hematoxylin–eosin stain, original magnification x40: showing an incipient horn pseudocyst. (C) Hematoxylin–eosin stain, original magnification x40: showing macrophages containing tattoo pigment in the superficial dermis.

A 4-mm punch biopsy was performed for diagnostic confirmation and to rule out verruca vulgaris. Histopathological analysis demonstrated epidermal acanthosis with mild papillomatosis, hyperkeratosis, and horn pseudocysts. Tattoo pigment and pigment incontinence were observed in the superficial dermis. No digitated papilomatosis, marked hypergranulosis, columnar parakeratosis, prominent capillary proliferation, focal capillary thrombosis, or a church-spire epidermal pattern were identified (Figure 2).

Human papillomavirus (HPV) genotyping was not performed, as this test is not routinely available in our public hospital setting. The combination of the clinical presentation, classic dermoscopic features, and unequivocal histopathological findings supported a final diagnosis of SKs. Given the entirely benign nature of the lesions and the absence of symptoms, the patient opted for conservative management without active intervention.

## Discussion

This case illustrates a rare instance of the Koebner-like phenomenon, with SKs arising exclusively within tattooed skin. Tattooing induces not only mechanical injury but also a sustained inflammatory response to exogenous pigment, which may provide an ongoing stimulus for epidermal proliferation [3,5,7,9]. While the Koebner phenomenon is well-established in inflammatory dermatoses, its occurrence in benign epidermal tumors such as SK remains exceptionally rare, with only a handful of cases documented [2-4,7,9]. This rarity is noteworthy, as the development of SK is typically spontaneous rather than trauma-induced [1,4,5,6,9,11]. 

The precise pathophysiology linking trauma to SK development is not fully elucidated [3,7]. It has been proposed that local trauma induces a pro-inflammatory milieu, triggering keratinocyte proliferation and epidermal hyperplasia through cytokine-mediated signaling (e.g., IL-1α, TNF-α) and oxidative stress [2,7,8]. In this patient, who has a history of pemphigus vulgaris, a condition itself known to exhibit koebnerization, an underlying predisposition to isomorphic responses may have been a contributing factor [3,7]. Furthermore, the persistent presence of exogenous tattoo pigment could act as a chronic local stimulus, perpetuating keratinocyte activation and aberrant epidermal differentiation [9,11].

A crucial diagnostic challenge lies in distinguishing SK from verruca vulgaris, as both can present as verrucous papules within tattoos [5,11-13]. Several reports highlight this pitfall: Lokhane et al. [13] and Eksomtramage et al. [12] highlighted that viral warts frequently mimic SKs clinically and dermoscopically, while Yuan et al. [1] and Bakke et al. [5] tattoo-localized SKs initially misdiagnosed as warts. This underscores the necessity for histopathological confirmation in atypical presentations.

Dermoscopy serves as a valuable non-invasive tool [2,11]. In our case, the classic cerebriform pattern with milia-like cysts was indicative of SK, contrasting with the thrombosed capillaries and papillomatous structures typical of verruca vulgaris [2,11]. Definitive diagnosis, however, rests on histopathology [2,5,6]. The findings of horn pseudocysts and acanthosis without marked papillomatosis and hypergranulosis, columnar parakeratosis, prominent capillary proliferation, and focal capillary thrombosis confirmed SK and ruled out an HPV-related lesion [2,5,6]. Notably, in similar reported cases, HPV testing has consistently yielded negative results, supporting the diagnosis of true, trauma-induced SK rather than a viral keratosis [5,6,11]. The key clinical, dermoscopic, and histopathological differences between SK and verruca vulgaris are summarized in Table 1.

**Table 1:** Diagnostic features distinguishing verruca vulgaris from seborrheic keratosis *Features may differ according to the post-traumatic interval and lesion evolution [2,5-6,11].

Diagnosis	Seborrheic keratosis	Verruca vulgaris
Clinical	Well-circumscribed, stuck-on appearance, waxy or verrucous surface, tan, brown, or black color, often exhibits keratin plugs (horn cysts)	Rough, hyperkeratotic papule/plaque, irregular surface, may have black dots, typically skin-colored or greyish
Dermoscopic	Cerebriform pattern (sulci and gyri), milia-like cysts and comedo-like openings, fissures and sharp demarcation, vessels absent or sparse (looped if present)	Dotted or glomerular vessels with white halo, red-black dots/streaks (thrombosed capillaries), irregular, verrucous surface, yellowish structureless areas
Histopathologic	Horn pseudocysts within the epidermis, basaloid proliferation, acanthosis, normal or slightly thickened granular layer, koilocytes: absent	Papillomatosis with inward-turning rete ridges, marked hypergranulosis and parakeratosis, koilocytes: present, dilated capillaries in dermal papillae

Accurate differentiation is critical for management. Unnecessary invasive procedures for a benign SK (e.g., cryotherapy, curettage) can lead to scarring or pigmentary changes [3,4]. Management of asymptomatic SK is typically observational; if treatment is desired for cosmetic reasons, modalities such as cryotherapy or shave excision are effective [2,9,14].

Our case, therefore, expands the clinical spectrum of the Koebner phenomenon to include SK induced by tattooing, highlighting the need for awareness of this uncommon presentation among dermatologists and pathologists.

## Conclusions

This case highlights a rare manifestation of the Koebner phenomenon in which SK developed exclusively within tattooed skin following cutaneous trauma. Although koebnerization is classically associated with inflammatory dermatoses, its occurrence in benign epidermal tumors such as SK is exceptional and likely underrecognized. This presentation represents an important diagnostic pitfall, as SK arising in tattoos may closely mimic verruca vulgaris, potentially leading to misdiagnosis and unnecessary treatment. Awareness of this uncommon entity, supported by dermoscopic and histopathological evaluation, is essential for accurate diagnosis and appropriate management.
